# Advances in Soil Fertility Management for Sustainable Crop Production

**DOI:** 10.3390/plants15010078

**Published:** 2025-12-26

**Authors:** Mohamed Ait-El-Mokhtar

**Affiliations:** Laboratory of Biotechnology, Agri-Food, Materials and Environment (LBAME), Department of Biology, Faculty of Science and Techniques, Mohammedia, Hassan II University of Casablanca, Mohammedia 28800, Morocco; mohamed.aitelmokhtar@univh2c.ma

There is an urgent need to develop more efficient, smart, and reliable fertilizers to boost soil fertility and to meet the increasing demand for agricultural products in a changing environment. Many creative approaches have surfaced in the pursuit of sustainable technologies to increase resilience and productivity. This Special Issue, “Advances in Soil Fertility Management for Sustainable Crop Production”, presents a collection of ground-breaking studies in an extensive array of disciplines, including the application of different strategies, technologies, and practices such as beneficial microorganisms, organic amendments, and intercropping. These strategies were applied in different formulations and in combination with reduced rates of classic chemical fertilizers to foster sustainable soil fertility and crop production. This Editorial seeks to contextualize the contributions of the featured papers and highlight a mosaic of information that presents new strategies to improve soil health and crop yield quantity and quality under different environmental conditions.

Many studies in this collection emphasize the impact of the application of organic amendment and/or beneficial microorganisms to promote soil fertility and crop production. Mitsigiorgi et al. [1] assessed the effects of various recycled organic substrates on lettuce performance and rhizosphere microflora. Almost all groups containing soil improvers demonstrated a significant increase in growth traits and chlorophyll content while displayed reduced stress marker concentrations. In addition, the few bioactive strains isolated from the amended substrates present moderate inhibition of *Bacillus subtilis* growth index. In another study, Jin et al. [2] examined how a soil conditioner (SC) containing halotolerant microbes affects rice quality, yield, soil fertility, and the physicochemical and structural characteristics of starch in hybrid rice grown in saline environments. By controlling the organic matter and nutritional content of the soil, the antioxidant defense system, and the source–sink connection, the application of this SC increased the yield and salt tolerance of hybrid rice, enhanced the rice’s appearance and flavor, and optimized the physicochemical characteristics of the rice starch. Marchão et al. [3] investigated the impact of adding plant-growth-promoting bacteria (PGPB) to post-emergence soybeans that had already received a *Bradyrhizobium* spp. inoculation. When sprayed on emerged soybeans, all treatments containing PGPB from the genera *Azospirillum*, *Pseudomonas*, *Priestia*, and *Bacillus* increased grain production, with grain yield increases being greatest in commercial formulations incorporating *Azospirillum brasilense*. Mekkaoui et al. [4] assessed tomato behavior under salt stress when treated with arbuscular mycorrhizal fungi (AMF) and compost. These authors revealed that the use of compost and AMF separately or in combination showed positive effects on photosynthetic apparatus, nitrogen (N) and osmoprotectants content, and antioxidant activity while lowering stress marker levels. In the same vein, Cai et al. [5] investigated the effect of microbial fertilizers and livestock manure organic fertilizer on oat agronomic traits, yield quality, and soil health in alpine ecosystems. The results showed that the co-application of both biofertilizers greatly enhanced soil nutrient content while promoting oat growth and development. According to these studies, applying beneficial microbes and organic amendments, separately or in combination, appears to be a successful strategy for improving crop performance and soil fertility under optimal and suboptimal conditions.

On the other hand, the combined application of conventional fertilizers with organic amendments seems to be one of the most effective strategies to boost soil health and crop fitness. For instance, Meng et al. [6] investigated the effects of glycolipid application in combination with a reduced N fertilizer on maize physiological and yield performance and N use efficiency through a three-year *in situ* field experiment. Their findings showed that the application of glycolipids with 20% reduced N fertilization maintained soil fertility, while the use of glycolipids with 10% N fertilizer reduction promoted plant growth and crop yield compared to the NPK treatment. In another study involving a two-year field experiment, Kakabouki et al. [7] studied the effects of different urea fertilizers and tomato pomace-based composts on the performance and quality traits of processing tomato and soil properties. The results revealed that the organic fertilization treatments (processing tomato pomace with farmyard manure (TP + FM), and processing tomato pomace with compost from plant residues (TP + CM)) showed the best effects on soil properties and tomato quality traits. In contrast, fruit yield and its components were significantly improved in urea with nitrification and urease inhibitors. Furthermore, Shao et al. [8] assessed the effect of potassium (K) fertilization coupled with biochar (BC) and cattle manure (CM) on the growth and yield of maize and soil physio-chemical characteristics in a two-year field experiment. This study’s findings demonstrated that by improving the physio-chemical properties of the soil, K fertilizer, in conjunction with BC and CM, enhanced growth and yield, with the combination of CM with K fertilizers generally proving to be more beneficial. Ma et al. [9], through a another two-year positioning experiment, demonstrated that optimized fertilization combining organic fertilizer and chemical fertilizer improved soil physicochemical traits and the activity of soil enzymes. The same fertilization increased the proportion of soil bacteria to the total number of microorganisms and maize yield. These studies imply that adequate chemical fertilizer application, in conjunction with organic amendments, may be a better choice for enhancing crop development and yield as well as soil fertility, which is essential for a sustainable agricultural output.

Jiang et al. [10] investigated the possibility of using *B. subtilis* with magnetic ionized water irrigation to reduce salinity. Their findings demonstrated that applying *B. subtilis* and using magnetized ionized water for irrigation, either separately or in combination, can successfully reduce soil pH and salt levels, increase microbial diversity and abundance, and raise cotton yield and quality, which provides effective and environmentally acceptable ways to increase cotton production capacity in saline soil.

The practice of intercropping could be another promising strategy to promote soil health and crop production. In an interesting study, Wu et al. [11] investigated the impact of maize and rice intercropping in dry farming on soil fertility and yield. When compared to a monoculture system, intercropping rice and maize under dry cultivation enhanced the overall development and yield, as well as multiple soil characteristics. As a result, plants in the intercropping system accumulated more phosphorus, K, and N. In addition, Zhang et al. [12] investigated the impact of sugarcane/soybean intercropping and reduced N application on organic carbon (SOC) sequestration. The study’s findings demonstrated that, in comparison to sugarcane monocropping (MS) treatment, sugarcane/soybean intercropping increased the C sequestration of sugarcane roots and shoots during the whole sugarcane growth period and soil labile organic C content and SOC content. This increased effect was more noticeable when N treatment was lowered.

Covering a different aspect of this Special Issue, Jadhav et al. [13] presented a review on the impact of microplastics (MPs) on different layers of plant function. The authors offer a thorough overview of the most recent research on the potential risks and negative effects of widespread microplastic occurrence in soil on crop production safety, including subjects pertaining to altered pesticide behavior and the spread of plant pathogens in the presence of MPs that may pose a threat to human health.

The articles included in this Special Issue collectively offer a patchwork of creative methods and discoveries that highlight the revolutionary potential of managing soil fertility in influencing sustainable crop production. The knowledge gained from this Special Issue provides important directions for promoting soil health, resilience, sustainability, and food security in agricultural systems around the world as we rationalize the use of the wide array of fertilizers available. We believe that these creative contributions will shed light, answer long-standing questions, and inspire researchers in critical areas pertaining to efficient innovative approaches tailored to sustainable soil fertility and crop production.
